# Goffin's cockatoos make the same tool type from different materials

**DOI:** 10.1098/rsbl.2016.0689

**Published:** 2016-11

**Authors:** Alice M. I. Auersperg, Stefan Borasinski, Isabelle Laumer, Alex Kacelnik

**Affiliations:** 1 Messerli Research Institute, University of Veterinary Medicine Vienna, Medical University of Vienna, University of Vienna, Veterinärplatz 1, 1210 Vienna, Austria; 2 Department of Cognitive Biology, University of Vienna, Althanstr 14, 1090 Vienna, Austria; 3 Department of Zoology, University of Oxford, OX1 3PS Oxford, UK

**Keywords:** innovation, tool manufacture, parrot

## Abstract

Innovative tool manufacture is rare and hard to isolate in animals. We show that an Indonesian generalist parrot, the Goffin's cockatoo, can flexibly and spontaneously transfer the manufacture of stick-type tools across three different materials. Each material required different manipulation patterns, including substrates that required active sculpting for achieving a functional, elongated shape.

## 1. Introduction

Animal tool use is a target of concerted research effort owing to its potential for revealing cognitive capabilities. For instance, the degree of flexibility shown by members of a species in making differently shaped tools, engaging in different manufacturing techniques and using different materials gives a measure of the relative contributions of heritable competence, individually and/or socially acquired skills and occurrences of true individual creativity ([1,2] for summaries). Here we focus on the manufacture of stick-type tools in the Goffin's cockatoo (*Cacatua goffiniana*) across a variety of materials.

Goffin's cockatoos do not build nests, nor are they known to be specialized for using foraging tools in the wild ([3]; M. O'Hara, B. Mioduszewska, D. Prawiradilaga, A. M. I. Auersperg, L. Huber, unpublished data, ongoing fieldwork). This suggests that tool related behaviours in this species are unlikely to express heritable predispositions for tool use, tool making or nest building as is the case in some corvids [4,5]. Their tool-related competence in the laboratory offers a valuable opportunity to isolate events of individual innovation. Research into Goffin's cockatoos' tool behaviour started after a captive male named Figaro spontaneously and reliably manufactured tools by cutting splinters out of larch wood, using them to rake in food placed behind the aviary grid [6]. In a set of 10 observations, Figaro showed nine instances of tool making, one involving a different substrate (snipping of a branch from a leafless twig). As he took approximately four times as long to make his first tool as for any subsequent tool it is likely that we recorded his original innovation event.

In a follow-up study, three males were able to emulate Figaro's tool use after receiving tool use (not manufacture) demonstrations [7]. Two later succeeded in making their own tools out of the same material (larch wood). One did so spontaneously and the other after one tool-making demonstration [7].

The substrate used in those experiments was larch wood. As the material breaks more easily along the age lines of the tree, it was unclear whether the elongated shape of the tools they made was accidental. Although the tools were of sufficient length and shape (i.e. slim enough to fit through the grid and long enough to reach the target) the animals might, for example, have bitten and torn the material out at random places, accidentally producing splinters that served as elongated, functional tools. To establish if the birds could actively produce an elongated shape, we confronted the four tool-using birds [7] with materials that required direct shaping owing to the absence of pre-existing structures, or that needed completely different manipulation patterns.

## 2. Material and methods

### (a) Subjects

Four hand-raised adult male Goffin's cockatoos were used. Three (Figaro, Dolittle and Kiwi) had previously sculpted tools out of larch wood. One (Figaro) had, on one occasion, also manufactured a tool by removing a side branch from a bamboo twig and another (Pipin) had used, but not made, tools before [6,7]. See electronic supplementary material, section A for more subject information.

### (b) Apparatus

**Figure 1.**
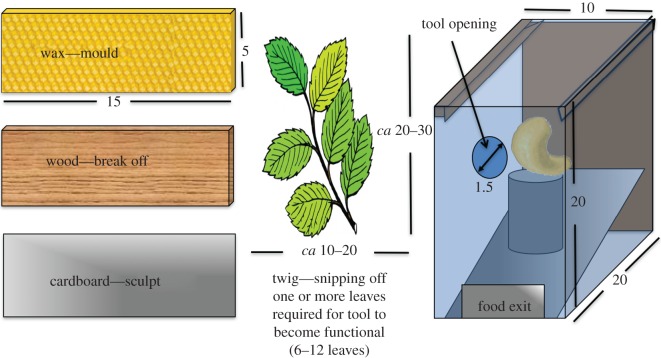
Left: material provided: beeswax (BW), larch wood (LW), cardboard (CB) or beech twig (TW). Right: apparatus, baited with a food reward. Dimensions are in centimetres.

The apparatus (figure 1) was a transparent box with a frontal hole (1.3 cm wide). One-sixth of a cashew nut rested on a platform inside. If the nut was knocked off the platform it would, consequently, slide out of the box through the frontal opening. An elongated object at least 6 cm long and thinner than the hole was required to agitate the nut out of its initial placing. All subjects had experience using ready-made 12 cm long round sticks with this apparatus [8].

Alongside the apparatus, we offered one of four different materials (figure 1): a block of larch wood (LW, 15 × 5 × 0.5 cm) a block of cardboard (CB, 15 × 5 × 0.3 cm, no longitudinal fibres), a block of natural beeswax with combs (BW, 15 × 5 × 0.5 cm) or a beech twig (TW), chosen to be relatively straight with at least six leaves 10–20 cm wide and 20–30 cm long (including leaves).

The testing conditions were defined by the materials. Subjects that had previously made tools from LW [6,7] started with this material; otherwise conditions were presented randomly across individuals (see electronic supplementary material, section B and table S1).

### (c) Procedure

The material was placed in front of the apparatus on an experimental table (1 × 1 m). The experimenter (S.B.) did not interfere in any way other than by replacing the material if the subject dropped it off the table. Trials stopped when the bird succeeded in retrieving the nut or failed to make a tool after 10 min elapsed.

Each condition consisted of up to five sessions of a maximum of 10 trials each. The full 10 trials were given only if the bird retrieved the food within 10 min on the previous trial. Subjects had to either complete two failure-free consecutive sessions (i.e. a total of 20 consecutive trials) or fail five consecutive sessions in order to move on to the next condition.

Trials were videotaped and pieces of material with which the birds touched the apparatus were measured and photographed. Data were collected during July–September 2014.

### (d) Analysis

Each trial was classified as success or failure. In successful trials, we scored the length and number of pieces used to touch the apparatus and scored manipulation time from the video files. Manipulation time could be split into manufacture time and tool use time for LW and CB. This separation was not possible for the TW as subjects repeatedly modified the partly inserted material. Hence, we scored the time from first touching the material to food retrieval.

Twenty per cent of the data was doubly scored (S.B. and I.L.). Inter-rater reliability was excellent (intraclass coefficient = 0.999; *F* = 3209; *p* < 0.001). Once a bird retrieved the reward consistently in 20 consecutive successful trials, we examined parameters of their behaviour along these trials. As electronic supplementary material, table S2 shows, these sequences started between 0 and 4 trials from the first exposure to the task. For the LW and CB tools, we used GLMMs with ‘tool length’, ‘manufacture time’ and ‘tool use time’ as target variables, ‘subjects’ as random effects and ‘material’ (LW/CB), ‘block’ (first 10 versus second 10 trials to test for learning effects) as fixed effects. We ran separate GLMMs for TW tools because for twigs ‘manipulation time’ could not be isolated, and the total length of the material was different for LW and CB before manipulation started. For this we used ‘group’ (the subjects that removed most or all leaves versus the subjects that removed few leaves) instead of ‘material’ as a fixed effect.

## 3. Results

### (a) Success

All subjects made TW tools, two of them being consistently successful from their first trial on. All three birds that had already made LW tools in a preceding study [7] remained successful from their first trial on and the bird that had previously failed to use LW continued to show an aversive response to the material. Two subjects succeeded in making CB tools, one being consistently successful from the first trial. No subject made tools out of BW. Individual success in tool making and tool extraction success is shown in the electronic supplementary material, section C, 1 and table S2.

### (b) Qualitative progress

#### (i) Larch wood

The birds bit laterally into the wood at the width of one age line (approx. 0.2–0.5 cm) and tore the resulting splinter off the block, ending up with an elongated shaped tool.

#### (ii) Beech twig

Two birds removed all or most of the leaves from their tools before inserting them, while the other two tended to only remove one to three leaves during insertions (see electronic supplementary material, section D). The two birds that failed to remove most leaves before their first insertion attempt repeatedly modified their tools after failed insertions, improving them progressively during use.

#### (iii) Cardboard

The two successful birds both placed a large number of parallel bite marks (CB tools: approx. 10 mm wide, see electronic supplementary material, section D) alongside the long edge of the material piece, cutting in a curve through the manufactured strip after reaching a certain length (see electronic supplementary material, section C, 4 and figure S4). The birds never modified LW and CB tools in successful trials but on a few occasions made a new tool after failing with a first one.

#### (iv) Beeswax

All made a few attempts to mould the wax, which resulted in useless segments which stuck to their beaks, and they soon lost motivation to interact with this material.

See electronic supplementary material, Movie S1 for examples of tool manufacture, and electronic supplementary material, section D for images of successful tools.

CB and TW tools were held at the proximal end for insertion through the hole and at the distal end while pushing them towards the reward (except in Figaro's case, who always held them by the distal end). Long TW tools with remaining leaves were inserted into the opening while adjusting the holding point.

### (b) Tool length

For LW and CB tools we detected an effect of ‘material’ on tool length but not for the other factors measured (see electronic supplementary material, section C, 3 and table S3).

**Figure 2.**
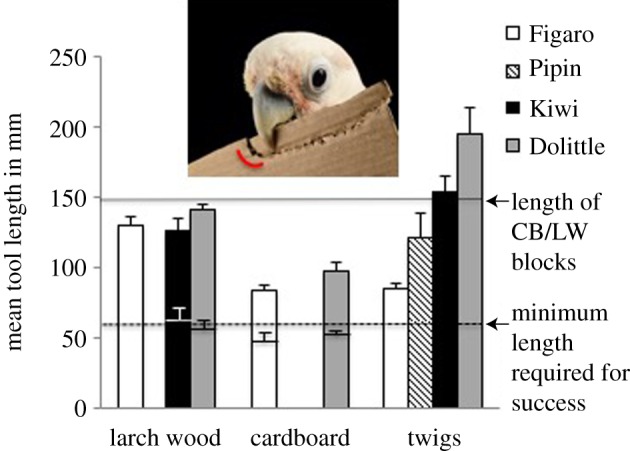
Average length of successful tools built in two consecutive successful sessions (20 trials) by each subject (bar shading) for each material (see labels on *x*-axis); error bars = s.e.; the second (lower) dataset within LW and CB represents the length of non-successful tools. Picture shows CB-tool making (picture credits: Solvin Zankl). (Online version in colour.)

CB tools were shorter than LW tools (figure 2): successful LW tools were almost the full length of the presented material, whereas CB tools were just about a centimetre longer than the absolute minimum length required to reach the reward (figure 2). Non-successful pieces were just below or close to the minimum length required to retrieve the food (figure 2; see also electronic supplementary material, section C, 2 and figure S1). We found an effect of ‘group’ in the GLMMs for TW tools (see electronic supplementary material, section C, 3 and table S4).

### (c) Manipulation time

Total manipulation time for TW tools tended to be shorter in the two birds that removed all or most leaves before insertion than in the other two (non-significant trend; figure 2; electronic supplementary material section C, 3 and figure S3 and table S4). Subject further showed a learning effect, with manipulation time decreasing between the first and second block in the TW condition (see electronic supplementary material, section C, 3 and figure S3 and table S4). Relative to LW, CB tools took longer to use but not to make. Tool use time for CB and LW also decreased from the first to the second block, revealing a learning effect (see electronic supplementary material section C, and table S3).

## 4. Discussion

Our results show that Goffin's cockatoos can make functional tools with similar shape from different materials, using distinctive manipulation patterns on the different substrates. Tested with materials with very different structure (fibrous larch wood, branched twigs and homogeneous cardboard), subjects showed fast and flexible transfer, with one bird being immediately consistently successful with three materials. The age lines of the larch made it easier to bite and then pull to split the wood along its own fault lines. In this case, the resulting pieces were almost the full length of the material blocks, much longer than needed and likely to be suboptimal in ergonomic terms. By contrast, cardboard tools were actively made by placing parallel bite-marks with the lower mandible, causing an effort penalty for excess length. Shredding of plant material (or paper in captivity) into strips has also been observed in nest-building lovebirds while collecting nesting material [9]. Nevertheless, such reports remain limited to nest-building species within a parrot genus that last shared an ancestor with Goffin's cockatoos *ca* 44 Ma (http://timetree.org). Furthermore, the cockatoos cut the pieces of cardboard close to the minimum length required to reach the food reward. After reaching the necessary length they curved the biting trajectory to cut, using the upper mandible. Thus, for cardboard, the shape left in the material block is a characteristic negative of the shape of the tool, similar to the shape left in pandanus tools after tool manufacture by New Caledonian crows [10]. Tools made from either cardboard or larch wood were seldom non-functional. If so, they were just below the minimum length required to retrieve the food. Further the animals finished the cutting of cardboard tools using a different behaviour. This indicates that they may control their actions relative to the required product, so that the length of their tool matches the distance at which the food is placed. Direct confirmation of this hypothesis requires future studies including manipulations of the required dimensions/properties of successful tools (i.e. length, width, flexibility).

The success in manufacturing tools from twigs further confirms that Goffin's cockatoos can make tools for a given function out of materials demanding very different manipulation patterns (snipping off excess material rather than removing the working piece). It is interesting that our birds tended to differ in the balance between effort and tool suitability. The two birds that removed fewer leaves took somewhat longer to obtain food with their twig tools, partly because their tools had to be further modified at a later stage. This may reflect inter-individual differences in impulsivity, as the birds may face a trade-off between starting to use a suboptimal tool or paying an upfront time cost in preparing a better one. A tantalizing possibility for future research in this or other species is that one may be able to predict which individual will make more elaborate tools from impulsivity data on inter-temporal choices in non-tool related tasks.

## Supplementary Material

Electronic supplementary methods and results

## Supplementary Material

Supplementary data

## Data accessibility

Data are available in the electronic supplementary material.
