# Supplementary material for: Goffin's cockatoos make the same tool type from different materials
Source: Biol Lett. 2016 Nov;12(11):20160689. doi: 10.1098/rsbl.2016.0689 (PMC5134049; doi:10.1098/rsbl.2016.0689)

Electronic Supplementary Material for:

Goffin's cockatoos make the same tool type from different materials

Alice M.I. Auersperg, Stefan Borasinski, Isabelle Laumer, Alex Kacelnik

A) Supplementary subjects information

Housing

The animals are permanently kept together in a social group of N=15 in a large aviary (indoors: 45m^2 ground space, 3-6m high wall to gable; outdoors: 150m^2 ground space, 3-5m high), enriched with branches, bathing puddles, hideaways and a variable selection of parrot toys (carefully selected to avoid experimental cueing). The indoor area is kept at 20 C° during wintertime. Food, consisting of a selection of seeds, fresh and dried fruit as well as various mineral and protein sources, and fresh drinking water are available ad libitum.

Sourcing of subjects and ethics:

All subjects were hand-raised and derive from accredited European breeders and have full CITES certificates. Our animals are not clipped and therefore partake in experiments voluntarily: they are called into the experimental chamber by name (we have not faced problems with the subjects' motivation to participate in any study for the past four years).

B) Order of conditions for each individual

Table S1 Order of conditions=1-4; C= Cardboard; L=Larch; T=Twig; W=Wax

	**ORDER**			
	**1**	**2**	**3**	**4**
Figaro	L	T	C	W
Pipin	T	C	W	L
Kiwi	L	C	W	T
Dolittle	L	W	T	C

C) Supplementary Results

1) Individual Success

Table S2 Individual succes across trials for each material

	Trial:	1	2	3	4	5	6	7	8	9	10	11	12	13	14	15	16	17	18	19	20	21	22	23
**Larch**	*Pipin*	□	□	□	□	□																		
	Figaro	■	■	■	■	■	■	■	■	■	■	■	■	■	■	■	■	■	■	■	■			
	Dolittle	■	■	■	■	■	■	■	■	■	■	■	■	■	■	■	■	■	■	■	■			
	Kiwi	■	■	■	■	■	■	■	■	■	■	■	■	■	■	■	■	■	■	■	■			
**Twig**	*Pipin*	□	1	□	■	■	■	■	■	■	■	■	■	■	■	■	■	■	■	■	■	■	■	■
	Figaro	■	■	■	■	■	■	■	■	■	■	■	■	■	■	■	■	■	■	■	■			
	Dolittle	■	■	■	■	■	■	■	■	■	■	■	■	■	■	■	■	■	■	■	■			
	Kiwi	□	□	□	3	■	■	■	■	■	■	■	■	■	■	■	■	■	■	■	■	■	■	■
**Card-board**	*Pipin*	□	□	□	□	□																		
	Figaro	■	■	■	■	■	■	■	■	■	■	■	■	■	■	■	■	■	■	■	■			
	Dolittle	□	□	2	■	■	■	■	■	■	■	■	■	■	■	■	■	■	■	■	■	■	■	■
	Kiwi	□	□	□	□	□																		
**Wax**	*Pipin*	□	□	□	□	□																		
	Figaro	□	□	□	□	□																		
	Dolittle	□	□	□	□	□																		
	Kiwi	□	□	□	□	□																		

■ Successful tool making in less than 10 minutes

□ Failed to make tools in 10 minutes

N N successes in less than 10 minutes, then failed to make one in 10 minutes

2) Number and dimensions of tools built

We also looked at the total number of material pieces that were combined with the apparatus, including unsuccessful tools for larch and cardboard (see Figure S1) for twigs this was not possible to evaluate clearly as subjects often modified pieces they had previously used instead of going back to the original source to create a new tool).

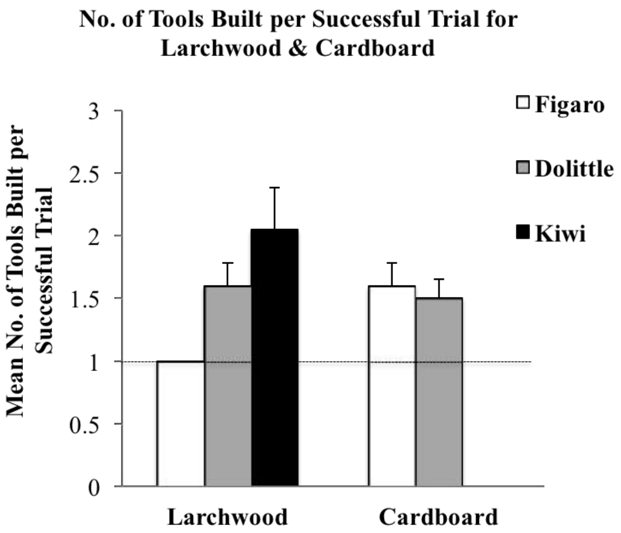

Figure S1 Mean no. of unsuccessful and successful tools built in the two consecutive successful sessions by each subject (bar colours) for each material (see labels on x-axis). Note that at least one tool is necessary. Eg. Figaro never built more than one LW tool. Error bars show SEs.

3) Tool Length & Manipulation time

Tool length descriptive

Dolittle's and Kiwi's successful Larch tools (Average length Dolittle successful = 14.1 cm +/-0.38 SE, non- successful = 5.68 cm +/-0.85 SE; Kiwi successful = 12.59 cm +/-0.97 SE, non-successful = 6.08 cm +/-1.18 SEM ; note that Figaro never built a non-successful Larch tool) and Dolittle, and Figaro's successful cardboard tools were on average longer than unsuccessful ones (Average length Dolittle successful = 9.75 cm +/- 0.67 SE, non-successful = 5.08 cm +/-0.37 SE; Figaro successful = 8.43 cm +/-0.35 SE, non-successful= 5.33 cm +/- 0.28).

Range of tool length overview:

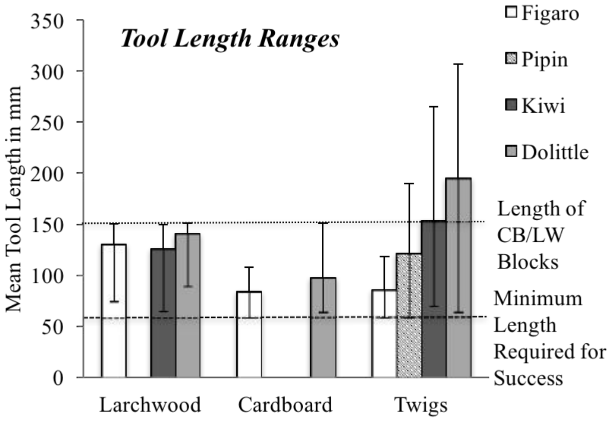

Figure S2 Mean length of successful tools in cm. The error bars show the full range of tool length for each material for each subject.

Manipulation times

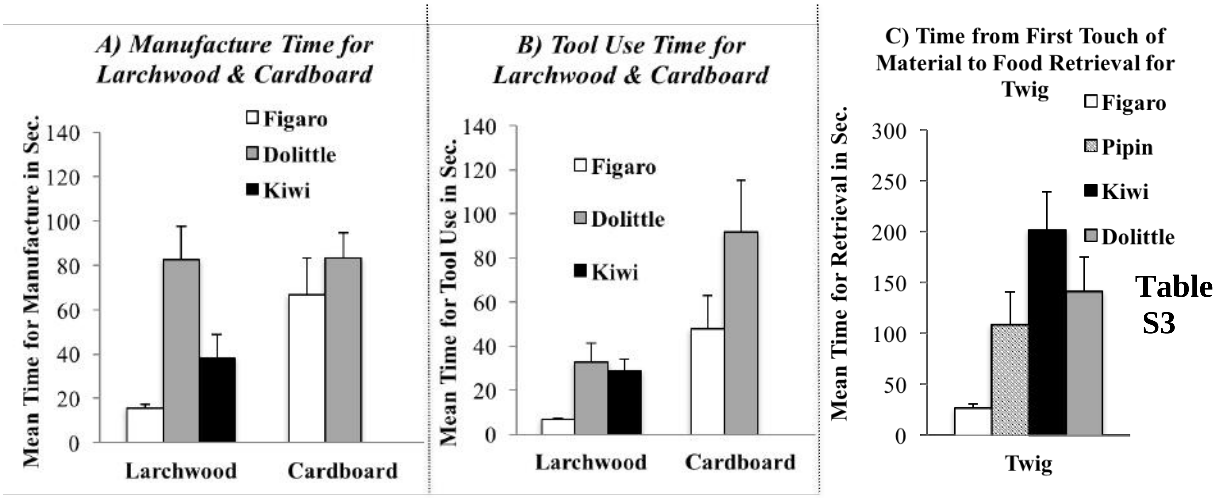

Figure S3 Mean time spent (in seconds) manufacturing cardboard and larch tools. 2) B Mean time spent using CB and LW tools. 2) C Mean time from first touching the material until the retrieval of the reward for TW tools. Error bars show SEs.

GLMM output Tool Length & Manipulation time

Table S3 Output GLMMs for the 20 consecutively successful Larchwood and Cardboard tools. Outcomes significantly above chance expectation are highlighted in grey.

**Fixed effects**		**Length**	**Manufacture time**	**Tool use time**
Material	*F*	33.138	3.093	29.513
	df 1	1	1	1
	df 2	7	7	7
	p	0.001	0.122	0.001
	Coefficient	42.764	-26.322	-49.459
	SE	7.429	14.966	9.104
Block	*F*	0.196	0.493	6.212
	df 1	1	1	1
	df 2	7	7	7
	p	0.671	0.505	0.041
	Coefficient	-3.133	9.7	-20.68
	SE	7.072	13.812	8.297

Table S4 Output GLMMs for the 20 consecutively successful Twig tools. Outcomes significantly above chance expectation are highlighted in grey, n.s. trends in light grey (p<0.1).

**Fixed effects**		**Manipulation time**	**Length**
Group	*F*	4.183	6.84
	df 1	1	1
	df 2	5	5
	p	0.096	0.047
	Coefficient	-104.15	-71.294
	SE	50.97	27.260
Block	*F*	10.698	1.2417
	df 1	1	1
	df 2	5	45
	p	0.022	0.278
	Coefficient	-80.05	-25.306
	SE	24.474	21.260

4) Qualitative Data

Cardboard Manufacture

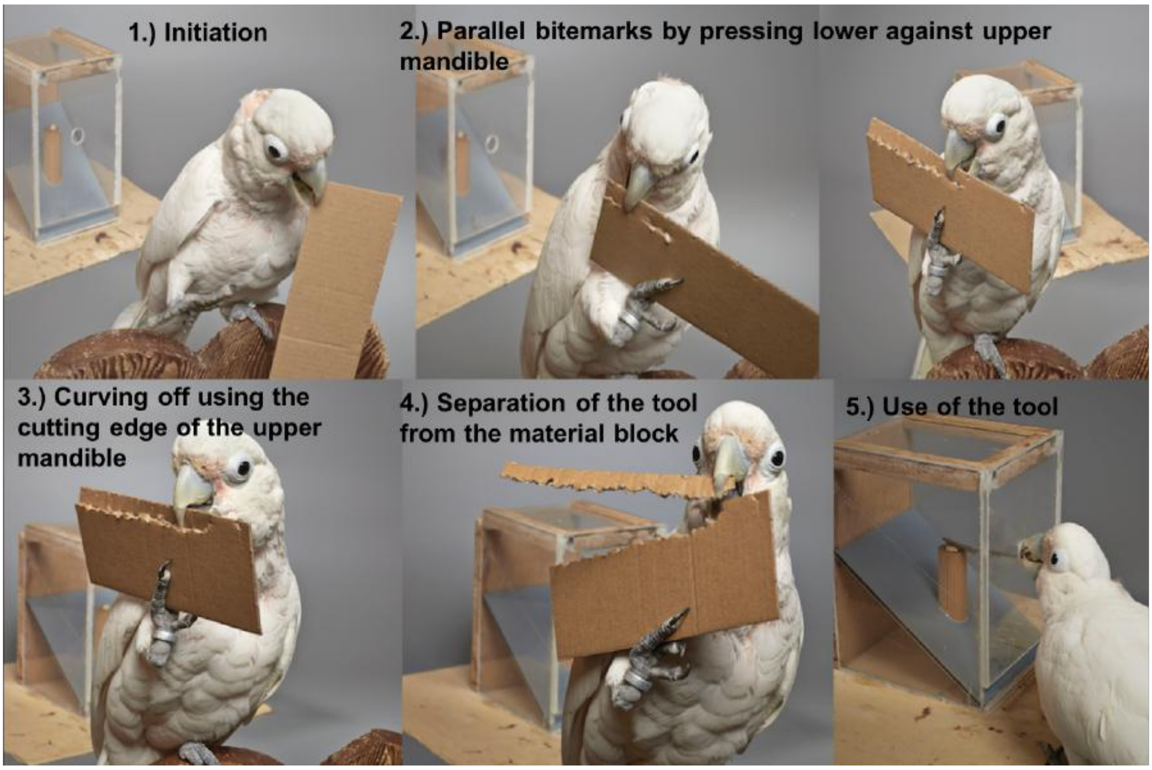

Figure S4 Sequence of manufacturing a tool from cardboard material. Picture credits: Bene Croy

D) Successful tools built in two 10-trial sessions following first success

Larchwood

Figaro

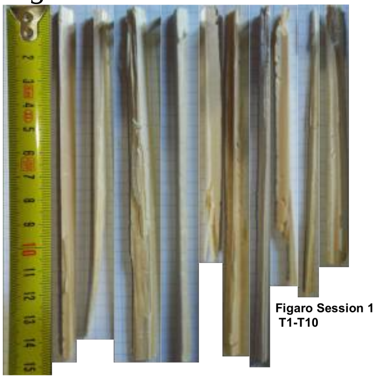

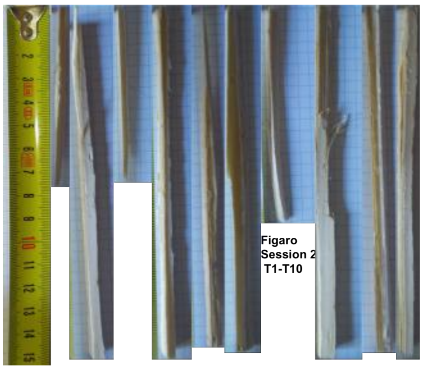

Dolittle

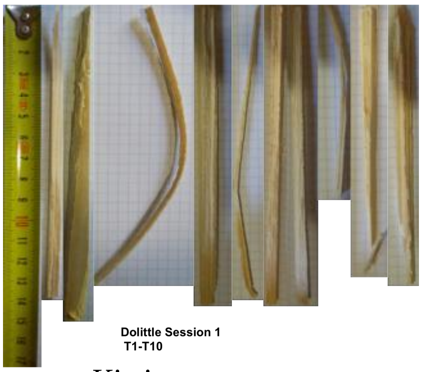

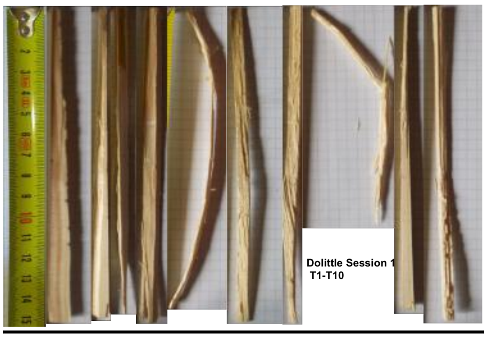

Kiwi

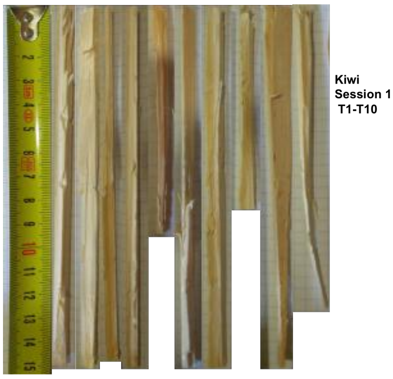

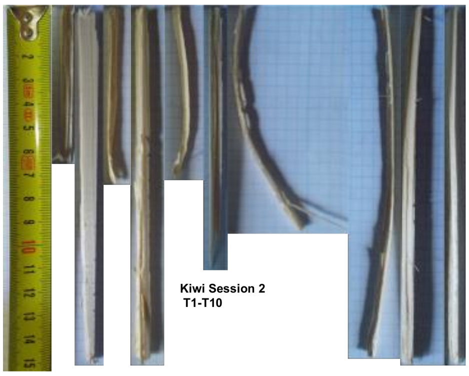

Cardboard

Dolittle

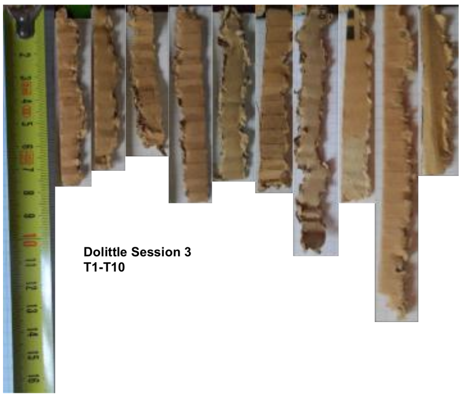

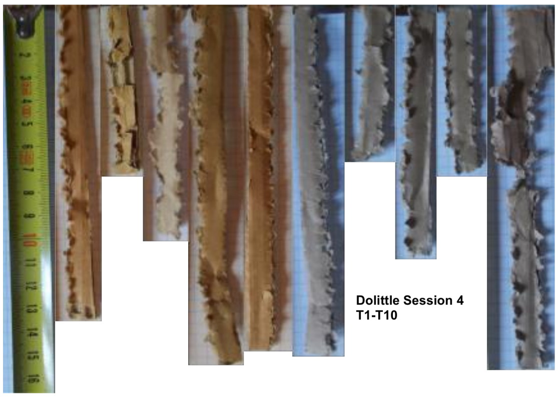

Figaro

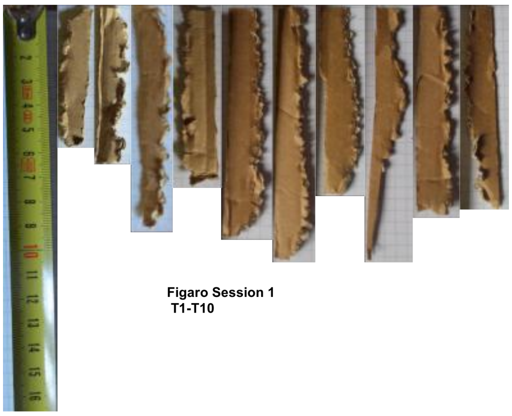

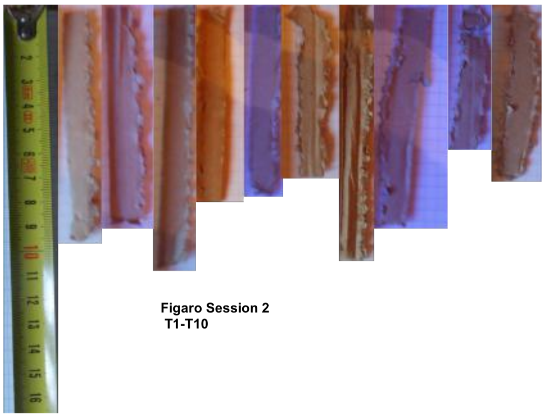

Twig

Figaro

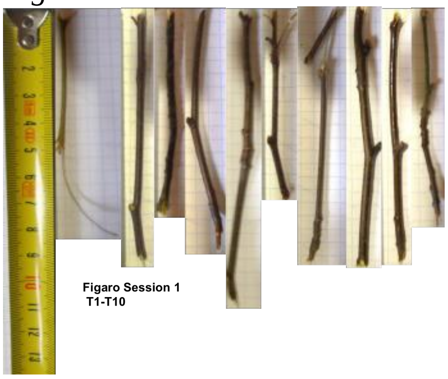

Figaro Session 1
T1-T10

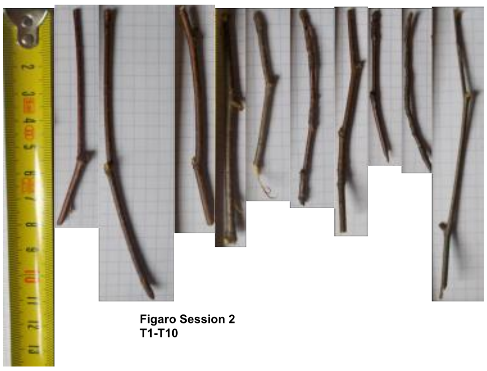

Figaro Session 2
T1-T10

Dolittle

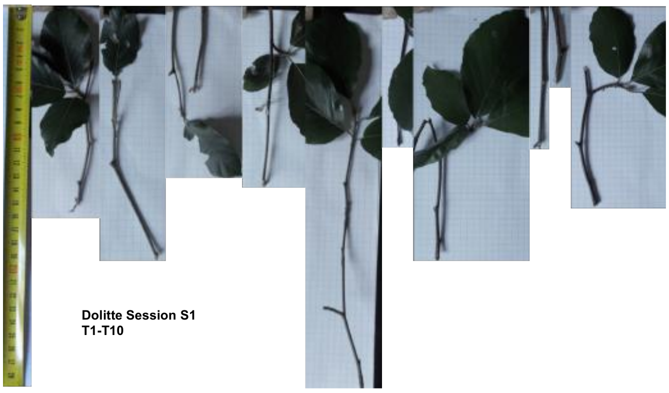

Dolitte Session S1
T1-T10

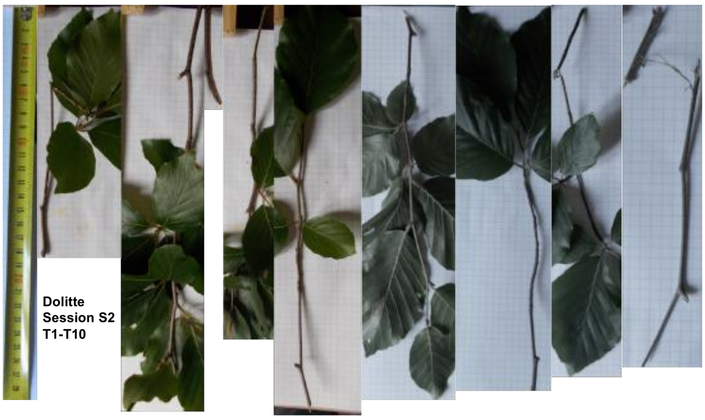

Dolitte
Session S2
T1-T10

Pipin

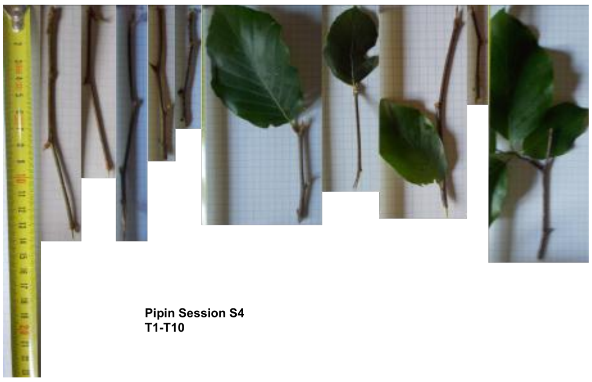

Pipin Session S4
T1-T10

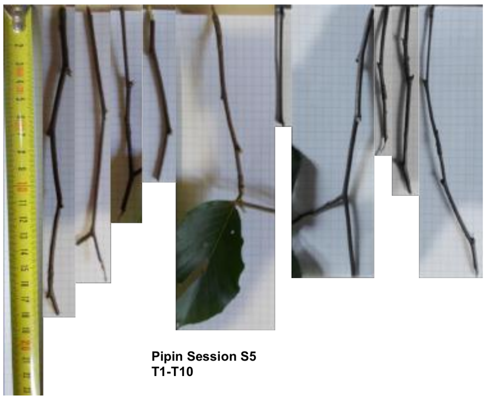

Pipin Session S5
T1-T10

Kiwi

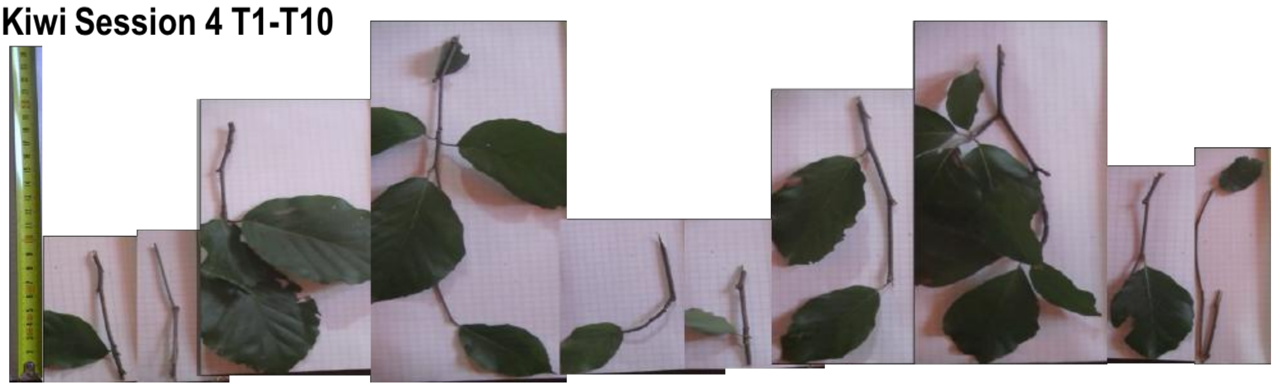

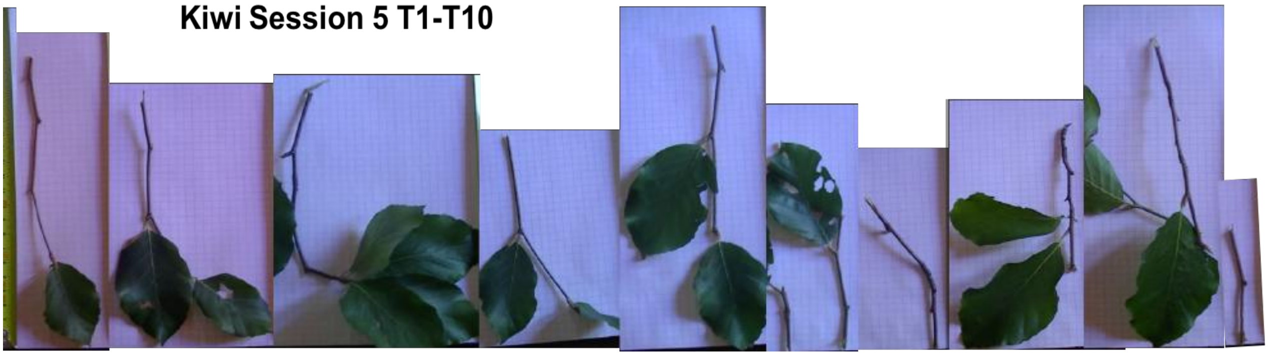

Supplement: Electronic supplementary methods and results [file rsbl20160689supp1.pdf]
